# Community Based Promotion on VCT Acceptance among Rural Migrants in Shanghai, China

**DOI:** 10.1371/journal.pone.0060106

**Published:** 2013-04-01

**Authors:** Tiejun Zhang, Xiuhong Tian, Fuchang Ma, Ying Yang, Feng Yu, Yanping Zhao, Meiyang Gao, Yingying Ding, Qingwu Jiang, Na He

**Affiliations:** 1 Department of Epidemiology, School of Public Health, Fudan University, and The Key Laboratory of Public Health Safety of Ministry of Education (Fudan University), Shanghai, China; 2 Minghang District Center for Disease Control and Prevention, Shanghai, China; Universitat Rovira i Virgili, Spain

## Abstract

**Objective:**

Voluntary counseling and testing (VCT) plays an important integral role in response to the HIV/AIDS epidemic. However, VCT service has not been effectively utilized among rural migrants, a high risk group in China. In this study, we developed a community based intervention to examine if community mobilization with comprehensive VCT is more effective than current HIV preventions with routine VCT service in promoting VCT acceptability among rural migrants in Shanghai, China.

**Methods:**

A comprehensive intervention with community mobilization and comprehensive VCT services including community-based VCT and mobile VCT was implemented during 2007–2009. Three communities in Minhang District of Shanghai were randomly selected and were designed to receive community mobilization and comprehensive VCT, traditional VCT and none intervention, respectively. After 24 months intervention, effects were evaluated by comparing outcome indicators between the baseline (2,690 participants) and follow-up surveys (1,850 participants).

**Findings:**

A substantial increase in VCT acceptance was observed among community mobilization group (94.9% vs. 88.5%, P<0.001), whereas the reverse effect was seen in the traditional VCT group (86.1% vs. 94.6%, P<0.001) and control group (69.0% vs. 91.7%, P<0.001). Rural migrants from community mobilization group were more likely to accept VCT (OR = 2.91, 95% CI 1.69–4.97). Rural migrants from community mobilization group also showed significant increase in HIV/AIDS knowledge, positive attitude towards HIV positive individuals and condom use.

**Conclusion:**

Community mobilization with comprehensive VCT has significant impact on promotion of VCT acceptance and utilization among rural migrants in Shanghai. These findings provide evidence to support community mobilization as a suitable strategy for VCT promotion among rural migrants in Shanghai, China.

## Introduction

Knowing one’s HIV status is the first step to accessing care and preventing further infection [1]. It may also serve as an important entry point for HIV-infected individuals to get access to further HIV-related care and treatment [2], [3]. However, among 700,000 people living HIV in China, the majority were still not identified [4].This underscores the urgent need for increasing acceptance and utilization of HIV testing especially voluntary HIV counseling and testing (VCT). Such need is obvious for rural migrants, since some previous studies suggest that rural migrants have played important roles in HIV transmission in China [5], [6], [7], [8], [9], [10], Limited education, constant mobility, hazardous working conditions, low wages, chronic underemployment, and substandard housing are also factors that can compromise the health of rural migrant laborers and make them more vulnerable to HIV [5], [9], [10], [11]. On the other hand, rural migrants are in danger of being marginalized and have no entitlement to urban health care and service accorded to most urban dwellers due to their non-urban residence [12].

During the past ten years, China has devoted huge efforts to promotion of free VCT service and established more than four thousand VCT clinics all over the country, where free and professional HIV counseling and testing are available [13]. This provides a unique opportunity for risk-taking persons to receive HIV counseling and testing service. Nonetheless, in a study by Liu and colleagues [14], 88.1% of 653 participants who had heard of HIV/AIDS said they would be willing to be tested. The actual rate of VCT use in that study was, however, not tested. Another study in China found that only 8.5% of participating premarital couples chose to accept HIV testing [15]. Moreover, Ma et al. found VCT uptake rate among general adults could be as low as 2.5% [16]. Stigma, discrimination and low HIV/AIDS knowledge were associated with low acceptance rates [17]. Similarly, our previous studies also showed that only a very small proportion (2.3%–3.4%) of rural migrants had ever had HIV testing with few had been tested at VCT clinics [5], [18], of which none was specifically designed and developed for rural migrants.

To fill this gap, a comprehensive community-based VCT promotion program featured with community mobilization had been conducted in Minhang district, Shanghai, China from 2007 to 2009. The central aim of the intervention was to test the assumption that compared with traditional and routine HIV prevention and VCT services, community mobilization for HIV prevention with multiple VCT services could significantly improve acceptance of VCT as well as HIV/AIDS knowledge and condom use among rural migrants.

## Materials and Methods

### Ethics Statement

This study was approved by the Institutional Review Board of Fudan University, China. Written consent was obtained from all the adult participants before any procedures were performed. For adolescents, informed written consent was obtained from the guardians on their behalf. Interview data did not bear the names of respondents and all data were anonymized before analysis.

### Study Sites

This study was conducted in Minhang District, one of the 19 districts of Shanghai, China. Shanghai is the most populous and economically developed city in China, with 17 million permanent residents and 5 million rural migrants. The first HIV case was reported in 1987. By the end of 2007, the number of reported HIV/AIDS cases in Shanghai was 2895, the majority of whom were rural migrants (Shanghai Center for Disease Prevention and Control, 2007). The real number of HIV/AIDS cases is probably much higher because of the lack of an active HIV surveillance system. The present study was executed in three communities in Minhang district with 288,000 officially registered local permanent residents (i.e., having “hu-kou” of Shanghai) and 220,000 registered rural migrants.

### Procedure

Briefly, a population based baseline survey was implemented among rural migrants in three selected communities in the year 2007, where participants were asked about their previous HIV testing experience and willingness to take VCT [18]. Then a two-year HIV/AIDS intervention program was introduced and implemented on the community level to promote VCT acceptance among rural migrants. The effectiveness of community mobilization on HIV/AIDS knowledge, attitude and behaviors as well as the acceptance of VCT was finally evaluated through a population based follow-up survey in 2009.

### Intervention Programme

**Figure 1:**
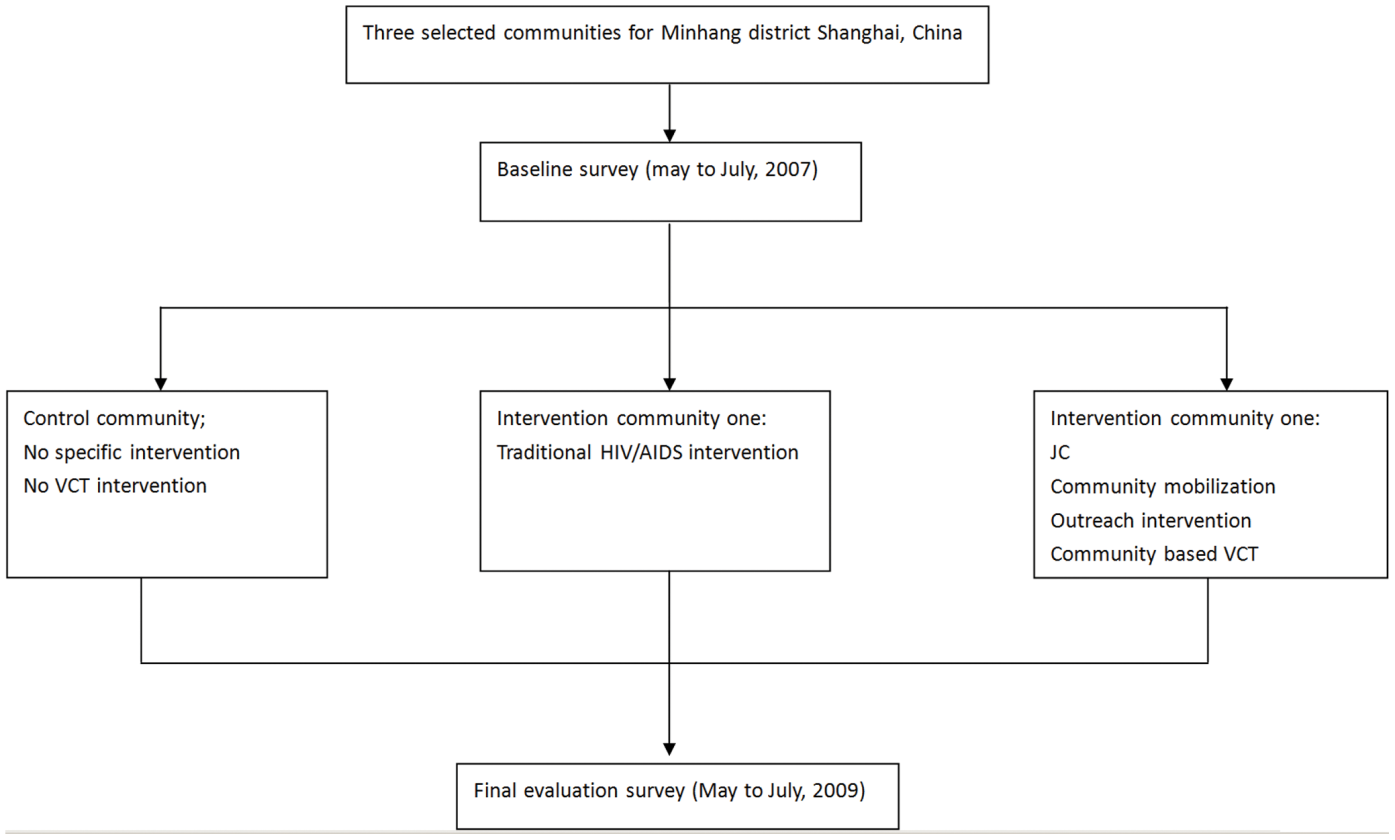
Schematic diagram of the community-based intervention program in the three selected communities.

As shown in figure 1, three out of a total of twelve township communities in Minhang district, including Qibao (QB), Xinzhuang (XZ) and Jiangchuan (JC) communities, were randomly selected for this study. QB community was designated as the control group, which was offered no intervention for VCT promotion (i.e., VCT service was not available in the community but was only available in the District Center for Disease Control and Prevention (CDC)). XZ and JC community were assigned to the experimental group; XZ, for the traditional model of VCT promotion (i.e., in addition to the VCT service available in the District CDC, VCT service was also available in the community by establishing a new VCT clinic in the community), and JC for community mobilization (CM) plus a comprehensive strategy for HIV testing and counseling (CM plus comprehensive VCT, including *establishing a new VCT clinic in the community, outreach mobile VCT and integration of VCT into family planning and specific clinics*). Each site had a full time coordinator who oversaw and facilitated the procedure.

Community mobilization used multiple methods to enhance the uptake of VCT and to reduce stigma, while comprehensive VCT service was designed to remove structural barriers (fees, inconvenience, and waiting for results). The community with CM plus comprehensive VCT, i.e., JC community was mobilized to set up a committee for HIV/AIDS prevention, which consisted of community leaders, gatekeepers, and community health workers. Committee was informed to recognize the problems of HIV/AIDS before project initiation. An intervention working group was established to implement the intervention and to make it consistent with the research protocol.

### Baseline and Follow Up Survey Procedure

To evaluate the effectiveness of the intervention program, two repeated cross-sectional surveys were conducted in the year 2007 and 2009, respectively. First, a baseline survey was conducted in the selected 3 communities during may to July 2007 to provide a baseline for selected outcome indicators. Enrollment of the study subjects have been reported previously [18]. Briefly, due to the large size of the rural migrant population in the study site and the lack of a sampling frame, a semi-random sampling approach called quota-sampling was employed for the sample selection. The quota sampling required that the number of subjects recruited from a specific venue category in the sample was proportional to the number of subjects employed in that specific venue category in the whole migrant population. From each venue category, rural migrants were recruited consecutively until the required number of study participants for that venue category was met. Totally, 2,832 rural migrants (about 3% of the migrant population in the study site) working in five broad venue categories were approached, and 2,690 (95.0%) of them finally agreed to participate in the baseline survey. During May to July 2009, a repeated follow-up survey using identical sampling procedures was carried out in the same 3 communities. A total of 1,962 rural migrants were approached, and 1,850 (94.2%) of them agreed to participate in the survey, 577 for QB, 839 for XZ and 434 for JC, respectively.

### Data Collection

A five-part anonymous questionnaire was developed to obtain information about socio-demographic characteristics, migration status, knowledge and attitudes concerning HIV/AIDS and infected individuals, sexual behaviors, and knowledge, attitude and practice (KAP) of VCT. HIV/AIDS knowledge was measured by eleven questions (Five questions were about the three transmission modes of HIV and condom use for HIV prevention, 4 regarding misconceptions about HIV transmission, 1 about HIV diagnosis, and 1 on whether HIV/AIDS was curable). The total score for HIV knowledge ranged from 0 to 11 (a score of 0 was designated for a wrong answer or an answer of unknown or unsure, and 1 for a correct answer). The majority of the questions have been widely used or reported in the literature. All instruments were pre-tested, and subsequent revisions were made. Interviews were administered face-to-face by trained public health workers. A small incentive equivalent to U.S. $3 was given to each participant for compensation of their time. The completed questionnaires were placed in a large black bag containing other completed questionnaires, reassuring the participants that no one could identify their answered questionnaires.

### Statistical Analysis

Descriptive analyses were conducted to illustrate KAP of HIV/AIDS and VCT for HIV. Changes in knowledge and attitude about HIV/AIDS, sexual behaviors, and voluntary HIV counseling and testing acceptance between surveys were compared based on the chi-squared test or Fisher’s exact test, whichever was appropriate. A multiple logistic regression analysis was conducted to identify factors independently associated with the willingness to take VCT. Their respective odds ratios (ORs) and 95% confidence intervals (CIs) were calculated. All statistical analyses were carried out using the SAS System for Windows (Cary, NC), Version 10.0. A p-value of less than 0.05 was deemed to be statistically significant.

## Results

### Characteristics of Study Participants

The socio-demographic characteristic of the participants in baseline and follow up surveys from three communities were shown in Table 1. Participants from control community showed significant difference in almost all the social and demographic characteristics between the baseline and the follow-up surveys, with only duration in Shanghai being comparable between the two surveys (P = 0.370). On the other hand, participants from both experimental communities were significantly different in some of the socio-demographic characteristics between the baseline and follow-up surveys. The profile of incomparable characteristic variables varied between the two experimental communities (Table 1).

**Table 1:** Sociodemographic characteristics of the baseline and follow-up survey participants*.

		Control community (QB)			Traditional VCTcommunity (XZ)			Community Mobilization plus comprehensive VCT community (JC)	
	Baseline No. (%)	Follow-up No. (%)	P	Baseline No. (%)	Follow-up No. (%)	P	Baseline No. (%)	Follow-up No. (%)	P
**Gender**			<0.001			0.203			0.443
Male	397 (34.1)	252 (43.7)		464 (47.5)	424 (50.5)		317 (57.7)	240 (55.3)	
Female	768 (65.9)	325 (56.3)		512 (52.5)	415 (49.5)		232 (42.3)	194 (44.7)	
**Age (years)**			<0.001			0.389			0.038
15–	466 (40.0)	230 (39.9)		323 (33.1)	275 (32.8)		267 (48.6)	237 (54.6)	
25–	425 (36.5)	167 (28.9)		404 (41.4)	324 (38.6)		169 (30.8)	124 (28.6)	
35–	233 (20.0)	135 (23.4)		199 (20.4)	198 (23.6)		99 (18.0)	55 (12.7)	
45–65	41 (3.5)	45 (7.8)		50 (5.1)	42 (5.0)		14 (2.6)	18 (4.1)	
**Education (School)**			<0.001			<0.001			<0.001
Primary or lower	191 (16.4)	60 (10.4)		140 (14.3)	60 (7.2)		83 (15.1)	29 (6.7)	
Middle	701 (16.4)	330 (57.2)		481 (49.3)	416 (49.5)		287 (52.3)	227 (52.3)	
High or college	273 (23.4)	187 (32.4)		355 (36.4)	363 (43.3)		179 (32.6)	178 (41.0)	
**Marital Status**			0.007			0.629			<0.001
Single	431 (37.0)	252 (43.7)		328 (33.6)	291 (34.7)		251 (45.7)	253 (58.3)	
Ever married	734 (63.0)	325 (56.3)		648 (66.4)	548 (65.3)		298 (54.3)	181 (41.7)	
**Working Venue**			<0.001			<0.001			0.124
Entertainment establishment	100 (8.6)	100 (17.3)		120 (12.3)	118 (14.1)		119 (21.7)	120 (27.6)	
Market	476 (40.9)	238 (41.3)		31 (3.2)	60 (7.2)		77 (14.0)	60 (14.1)	
Factory	330 (28.3)	165 (28.6)		331 (33.9)	299 (35.5)		182 (33.2)	120 (27.6)	
Community service	229 (19.7)	44 (7.6)		385 (39.5)	293 (35.0)		82 (14.9)	73 (16.8)	
Construction site	30 (2.6)	30 (5.2)		109 (11.2)	69 (8.2)		89 (16.2)	61 (13.9)	
**Duration in Shanghai (Months)**			0.370			<0.001			<0.001
<6	147 (12.6)	61 (10.6)		138 (14.1)	86 (10.3)		180 (32.8)	71 (16.4)	
6–11	81 (7.0)	39 (6.8)		80 (8.2)	53 (6.3)		47 (8.6)	49 (11.2)	
12–35	289 (24.8)	132 (22.8)		237 (24.3)	166 (19.8)		116 (21.1)	104 (24.0)	
36–	648 (55.6)	345 (59.8)		521 (53.4)	534 (63.6)		206 (37.5)	210 (48.4)	
**Living with spouse or a sexual partner**			0.027			0.060			0.581
No	546 (46.9)	238 (41.2)		405 (41.5)	385 (45.9)		176 (32.1)	132 (30.4)	
Yes	619 (53.1)	339 (58.8)		571 (58.5)	454 (54.1)		373 (67.9)	302 (69.6)	
**Number of migrated cities including Shanghai**			0.020			0.400			0.252
1	644 (55.3)	285 (49.4)		480 (49.2)	396 (47.2)		277 (50.5)	203 (46.8)	
> = 2	521 (44.7)	292 (50.6)		496 (50.8)	443 (52.8)		272 (49.5)	231 (53.2)	

* Chi-squared test or Fisher's exact test was applied when necessary. P<0.05 was regarded as significant.

### Changes in Knowledge and Attitude About HIV/AIDS

Knowledge and attitude about HIV/AIDS between the baseline and follow-up surveys in all the three communities were shown in Table 2. Comparisons of HIV/AIDS knowledge and attitudes towards HIV+ individuals (data not shown) among study participants at baseline yielded no significant differences between the control and experimental communities. However, compared with the baseline survey, rural migrants from both experimental communities showed higher HIV/AIDS knowledge level in the post-intervention follow-up survey, for example, the highest HIV/AIDS knowledge group increased from 38.3% to 83.2% (P<0.001) in JC, and from 35.8% to 46.1% (P<0.001) in XZ, whereas rural migrants in the control community showed lower level of HIV/AIDS knowledge in the post-intervention follow-up survey, with the proportion of having lowest level of HIV/AIDS knowledge increased from 14.2% to 21.3% (P<0.001).

**Table 2:** HIV/AIDS-related knowledge, attitude and sexual behavior among survey paritcipants before and after the intervention programme*.

		Control community(QB)			Traditional VCT community (XZ)			Community Mobilization plus comprehensive VCT community (JC)	
	Baseline	Follow-up	P	Baseline	Follow-up	P	Baseline	Follow-up	P
**HIV/AIDS knowledge (Score)**			<0.001			<0.001			<0.001
0–5	166 (14.2)	123 (21.3)		153 (15.7)	108 (12.9)		85 (15.4)	11 (2.5)	
6–8	593 (50.9)	287 (49.7)		474 (48.5)	344 (41.0)		254 (46.3)	62 (14.3)	
9–11	406 (34.9)	167 (28.9)		349 (35.8)	387 (46.1)		210 (38.3)	361 (83.2)	
**Willing to work with an HIV+ individual**			0.046			0.299			<0.001
No	708 (60.8)	379 (65.7)		530 (54.3)	476 (56.7)		320 (58.3)	117 (27.0)	
Yes	457 (39.2)	198 (34.3)		446 (45.7)	363 (43.3)		229 (41.7)	317 (73.0)	
**Having ever had sexual intercourse**			0.044			0.199			0.242
No	278 (23.9)	113 (19.6)		172 (17.6)	129 (15.4)		143 (26.1)	99 (22.8)	
Yes	887 (76.1)	464 (80.4)		804 (82.4)	710 (84.6)		406 (73.9)	335 (77.2)	
**Having had sex in the past month** **			0.043			0.557			<0.001
No	120 (13.5)	82 (17.7)		166 (20.6)	138 (19.4)		50 (12.5)	81 (24.2)	
Yes	767 (86.5)	382 (82.3)		638 (79.4)	572 (80.6)		351 (87.5)	254 (75.8)	
**Condom use among those having had sex in the past month**			<0.001			<0.001			<0.001
Always	110 (14.3)	47 (12.3)		156 (24.5)	133 (23.3)		68 (19.4)	71 (28.0)	
Sometimes	134 (17.5)	132 (34.6)		118 (18.5)	163 (28.5)		89 (25.4)	84 (33.1)	
Never	523 (68.2)	203 (53.1)		364 (57.1)	276 (48.2)		194 (55.3)	99 (39.0)	
**Purpose of using condoms among those having ever used a condom in the past month (with multiple answers)**									
To prevent dirty sex	53 (21.7)	24 (13.4)	0.029	49 (17.9)	44 (14.8)	0.313	23 (14.7)	25 (16.1)	0.735
To prevent pregnancy	188 (77.0)	143 (79.9)	0.484	223 (81.4)	230 (77.2)	0.216	118 (75.6)	128 (82.6)	0.132
To prevent diseases	68 (27.9)	49 (27.4)	0.911	103 (37.6)	109 (36.6)	0.802	65 (41.7)	84 (54.2)	0.027
**Number of sex partners in the past year among those who reported having had sex in the past year**			<0.001			0.012			0.482
1	815 (94.0)	393 (88.1)		701 (88.8)	631 (92.7)		342 (86.6)	272 (84.7)	
> = 2	52 (6.0)	53 (11.9)		88 (11.2)	50 (7.3)		53 (13.4)	49 (15.3)	

* Chi-squared test or Fisher's exact test was applied when necessary. P<0.05 was regarded as significant.

** With missing data.

The proportion of rural migrants willing to work with HIV+ individuals in JC community was significantly higher after intervention than before (73.0% vs. 41.7% P<0.001), whereas rural migrants in QB, the control community, were more reluctant to work with HIV+ individuals at the follow-up than the baseline (39.2% vs. 34.3%, P = 0.046).

### Changes in Sexual Behaviors among Study Participants

*Sexual life* There were 2,097 (77.9%) and 1,509 (81.6%) participants reported having lifetime sexual intercourse in the baseline and follow up surveys, respectively. The majority of them had sex in the past month. Compared with the baseline survey, the percentage of having sex in the past month among rural migrants in the follow-up survey was significantly lower in the control community and one experimental community (JC) (Table 2). About 41.3% and 58.4% of singles reported having had sex intercourse in the baseline and the follow-up surveys, respectively, without significant differences between three communities in both surveys (data not shown).

*Condom use* At the baseline, the rate of consistent condom use in the past month was 14.3%, 24.5% and 19.4%, and the rate of never using condoms was 68.2%, 57.1% and 55.3% for QB, XZ and JC, respectively (Table 2). At the follow-up, the rate of consistent condom use in the past month was 12.3%, 23.3% and 28.0% and the rate of never using condoms was 53.1%, 48.2% and 39.0% for QB, XZ and JC, respectively (Table 2). As shown in Table 2, a significant increase of condom use observed among rural migrants in the intervention communities especially JC community with community mobilization. Moreover, a significantly higher proportion of rural migrants in JC, the community with community mobilization plus comprehensive VCT, reported using condoms primarily for prevention of disease (54.2% vs. 41.7%, P = 0.027, Table 2). Single rural migrants used condoms more frequently than ever married rural migrants (data not shown).

*Multiple sex partners* Among those having had life time sexual intercourse, the proportion of participants having had multiple sex partners was 9.2% overall for the baseline survey and 10.0% overall for the follow-up survey. It had no significant change before and after the intervention in JC community, but had significant changes before and after the intervention in QB and XZ community. After the intervention program, the proportion of having had multiple sex partners was significantly increased in QB, the control community (QB) (11.9% vs.6.0%, P<0.001), and was significantly decreased (7.3% vs. 11.2%, P = 0.012) in XZ community (Table 2).

### Changes in Voluntary Counseling and Testing Acceptance

*Knowledge of VCT* Approximately a half of the rural migrants from QB community had never heard about VCT in either survey (45.6% and 49.6%, respectively, P = 0.117). In the experimental communities, the proportion of rural migrants having heard about VCT was significantly increased after intervention (85.3% vs. 48.1%for JC, P<0.001; and 56.4% vs. 46.8% for XZ, P<0.001) (Table 3).

**Table 3:** Knowledge, attitude, and practice of voluntary HIV counseling and testing and acceptance of HIV mass screening among survey participants*.

		Control community (QB)			Traditional VCT community (XZ)			Community Mobilization plus comprehensive VCT community (JC)	
	Baseline	Follow-up	P	Baseline	Follow-up	P	Baseline	Follow-up	P
**Had heard about VCT**			0.117			<0.001			**<0.001**
No	634 (54.4)	291 (50.4)		519 (53.2)	366 (43.6)		285 (51.9)	64 (14.7)	
Yes	531 (45.6)	286 (49.6)		457 (46.8)	473 (56.4)		264 (48.1)	370 (85.3)	
**Willing to take VCT upon perception of HIV risk**			<0.001			<0.001			**<0.001**
No	97 (8.3)	179 (31.0)		53 (5.4)	117 (13.9)		63 (11.5)	22 (5.1)	
Yes	1068 (91.7)	398 (69.0)		923 (94.6)	722 (86.1)		486 (88.5)	412 (94.9)	
**Ever had HIV test**			0.077			0.585			**<0.001**
No	1153 (99.0)	565 (97.9)		940 (96.3)	811 (96.8)		535 (97.5)	392 (90.3)	
Yes	12 (1.0)	12 (2.1)		36 (3.7)	28 (3.2)		14 (2.5)	42 (9.7)	
**Ever had HIV test in a VCT site**			0.292			0.574			0.085
No	1163 (99.8)	575 (99.7)		965 (98.8)	831 (99.0)		543 (98.9)	423 (97.5)	
Yes	2 (0.2)	2 (0.3)		11 (1.2)	8 (1.0)		6 (1.1)	11 (2.5)	
**Perceived risk of HIV infection**			0.020			0.034			**<0.001**
No	1000 (85.8)	466 (80.7)		847 (86.8)	705 (84.0)		439 (80.0)	182 (41.9)	
Not sure	130 (11.2)	84 (14.6)		105 (10.8)	95 (11.3)		74 (13.5)	34 (7.8)	
Yes	35 (3.0)	27 (4.7)		24 (2.5)	39 (4.6)		36 (6.6)	218 (50.3)	
**Acceptance of community-based, confidential and free mass HIV screening**			0.839			0.541			**<0.001**
No	235 (20.2)	114 (19.8)		134 (13.7)	107 (12.8)		98 (17.9)	30 (6.9)	
Yes	930 (79.8)	463 (80.2)		842 (86.3)	732 (87.2)		451 (82.1)	404 (93.1)	
**Acceptance of “opt-out” HIV testing in various clinical settings** **			0.082			0.252			**<0.001**
No	139 (11.9)	86 (14.9)		75 (7.7)	77 (9.2)		70 (12.8)	20 (4.6)	
Yes	1026 (88.1)	491 (85.1)		901 (92.3)	762 (90.8)		479 (87.2)	414 (95.4)	

* Chi-squared test or Fisher's exact test was applied when necessary. P<0.05 was regarded as significant.

** Including but not limited to general hospitals and clinics, antenatal clinics, physical examinations for employment or marriage license.

*HIV risk perception and acceptance of VCT* Very few participants (3.0% for QB, 2.5% for XZ and 6.6% for JC) felt that they were likely to be HIV-infected now or in the future before intervention, including those engaging in multiple sexual partnership without using condoms. Such low awareness had been significantly improved in JC community after community mobilization and comprehensive VCT promotion (50.3% vs.6.6%, P<0.001), while only slight increase, although significant, was observed in QB (4.7% vs. 3.0%, P = 0.020) and XZ (4.6%vs. 2.5%, P = 0.034) (Table 3).

Compared with the baseline survey, the proportion of participants willing to take VCT upon self-perception of HIV risks was lower at the follow-up survey in the control community (QB) (69.0% vs. 91.7%, P<0.001) and the community with traditional VCT (XZ) (86.1% vs. 94.6%, P<0.001), but was significantly higher in the community with community mobilization plus comprehensive VCT services (JC) (94.9% vs. 88.5%, P<0.001) (Table 3).

Based on the follow-up survey, a logistic regression analysis controlling for potential confounding variables indicated that community mobilization plus comprehensive VCT promotion, working venue, higher HIV/AIDS knowledge, being willing to work with HIV+ individuals, having heard about VCT, having ever had HIV test and having received HIV intervention were independently correlated with willingness to take VCT (Table 4).

**Table 4:** Multivariate logistic regression analysis of predictors for VCT acceptability among the follow-up survey participants* (n = 1850).

	Proportion of willingness to uptake VCT (%)	Adjusted OR (95%CI)	P value
**Intervention group**			
Control group (QB)	398/577 (69.0)	1.00	
Traditional VCT group (XZ)	722/839 (86.1)	1.84 (1.34–2.51)	**<0.001**
Community Mobilization group (JC)	412/434 (94.9)	2.91 (1.69–4.97)	**<0.001**
**Gender**			
Male	786/916 (85.8)	1.00	
Female	746/934 (79.9)	0.94 (0.68–1.30)	0.719
**Age (years)**			
15–25	603/742 (81.3)	1.00	
26–35	526/615 (85.5)	0.99 (0.67–1.49)	0.990
36–45	320/388 (82.5)	1.02 (0.61–1.69)	0.949
46–62	83/105 (79.0)	0.76 (0.38–1.50)	0.426
**Working Venue**			
Entertainment establishment	271/338 (80.2)	1.00	
Market	256/358 (71.5)	0.89 (0.68–1.72)	0.742
Factory	497/584 (85.1)	1.54 (0.95–2.50)	0.081
Community service	358/410 (87.3)	1.84 (1.12–2.97)	**0.015**
Construction site	150/160 (93.8)	4.34 (1.83–10.30)	**<0.001**
**Education (School)**			
Primary or lower	110/149 (73.8)	1.00	
Junior high	792/973 (81.4)	1.33 (0.82–2.14)	0.251
Senior high or college	630/728 (86.5)	1.55 (0.91–2.63)	0.106
**Marital status**			
Single	646/796 (81.2)	1.00	
Ever married	886/1054 (84.1)	1.21 (0.76–1.90)	0.422
**Resided in Shanghai (months)**			
<6	172/218 (78.9)	1.00	
7–12	119/141 (84.4)	1.59 (0.86–2.94)	0.138
13–36	331/402 (82.3)	1.30 (0.82–2.07)	0.268
37–	910/1089 (83.6)	1.31 (0.85–2.01)	0.228
**Living with spouse or a sexual partner**			
No	908/1095 (82.9)	1.00	
Yes	624/755 (82.6)	0.79(0.56–1.12)	0.182
**HIV/AIDS knowledge (score)**			
0–5	144/242 (59.5)	1.00	
6–8	555/693 (80.1)	2.33 (1.65–3.30)	**<0.001**
9–11	833/915 (91.0)	3.13 (2.23–4.93)	**<0.001**
**Willing to work with HIV(+) individual**			
No	741/972 (76.2)	1.00	
Yes	791/878 (90.0)	1.54 (1.14–2.07)	**0.005**
**Had heard about VCT**			
No	537/721 (74.5)	1.00	
Yes	995/1129 (88.1)	1.44 (1.09–1.91)	**0.010**
**Ever had HIV test**			
No	1451/1768 (82.1)	1.00	
Yes	81/82 (98.8)	9.91 (1.34–73.23)	**0.025**
**Perceived risk of HIV infection**			
No	1097/1353 (81.1)	1.00	
Not sure	167/213 (78.4)	0.96 (0.65–1.43)	0.862
Yes	268/284 (94.4)	1.69 (0.86–3.36)	0.129
**Received HIV program**			
No	904/1176 (76.9)	1.00	
Yes	628/674 (93.2)	1.76 (1.21–2.55)	**0.003**

* Odds Ratios (OR) adjusted for other variables listed in this table by a logistic regression analysis. CI: confidence interval.

*Practice of HIV testing and VCT* The proportion of rural migrants who ever had HIV test was significantly increased in JC community after intervention (9.7% vs. 2.5%). A marginally significant increase in the proportion of having HIV test in VCT sites was also reported among participants from JC community (2.5% vs. 1.1%) (Table 3). However, no significant difference in HIV testing practice was found in XZ and QB community after the intervention program (Table 3).

On the other hand, during the past 12 months before the follow-up survey, the proportion of VCT attendants who were rural migrants, the targeted population of the present intervention program, was 82.8% (87/105) in JC community, much higher than that in XZ (68.5%, or 37/54) and QB (62.5%, or 25/40) (P = 0.010).

*Acceptance of alternative HIV testing approaches* Compared with the baseline, both the acceptance community-based confidential and free mass HIV screening program (93.1% vs. 82.1%, P<0.001) and the acceptance of “opt-out” HIV testing in various clinical settings (95.4% vs. 87.2%, P<0.001) was significantly increased in JC community after the intervention. No differences were observed for the other two communities in the acceptance of alternative HIV testing approaches before and after the intervention (Table 3).

## Discussion

Despite the importance of VCT service and a substantial body of research into acceptability and practice of VCT in China mainland [5], [16], [19], [20], [21], [22], there have been few evaluations of programs to promote VCT uptake at a community level. To our knowledge, this is the first community-based intervention program intended to improve VCT-related knowledge, attitude and acceptance among rural migrants in metropolitan areas in China. The obtained information can be used to develop more effective VCT services targeting rural migrants in China. In addition, this study also augments the limited data available on HIV/AIDS-related knowledge, attitudes and sexual behavior among rural migrants in metropolitan areas in China, which is critical for developing effective HIV prevention and intervention programs.

Community based intervention has been successfully conducted to increase HIV testing in some other countries [23], [24], [25]. Data from this study also suggest that obvious and positive intervention effects of knowledge, attitude and practice concerning HIV/AIDS could be detected on the respondents from experimental communities, especially the community with community mobilization, in which rural migrants showed high awareness of HIV risks and high acceptance and practice of VCT after intervention. Nonetheless, although previous studies have shown that self-perceived risk of HIV infection is a determinant of VCT acceptability, data from this study did not reconfirm this result. A possible explanation is that participants who did not perceive HIV risk would also like to take a VCT service to learn their HIV infection status and to get more knowledge about HIV/AIDS prevention. On the other hand, the present study showed that although community mobilization could enhance VCT acceptance, such acceptance is not always equal to action. This kind of disparity between intention to take VCT and actual uptake of VCT was also observed in other studies in China [15], [16]. Taking VCT for HIV might be a complex behavior that is driven by many factors including but not limited to the intention to do so [26], [27].

This study further confirms that knowledge, attitudes and risk awareness towards HIV/AIDS could be significantly improved via implement of community mobilization with comprehensive HIV/AIDS intervention. Community mobilization may help to disseminate HIV/AIDS knowledge among rural migrants as previous reports [28], [29].

In the present study, rural migrants from XZ, the community with traditional VCT service, remained no significant changes in most outcome indicators especially knowledge, acceptance and practice of VCT over the 2 years, suggesting that the traditional mode of VCT service might be ineffective in promoting VCT acceptability and practice among rural migrants. Moreover, a slightly reverse effect of the intervention in condom use (24.5% vs. 23.3%) and willingness of VCT uptake (94.6% vs. 86.1%) between the baseline and follow-up surveys were observed among participants from the traditional VCT group. It suggests the weakness of the traditional intervention in VCT promotion. This further underscores the importance of community mobilization in HIV prevention and intervention programs especially in VCT promotion programs.

Concerns about confidentiality and stigma have been found to be important factors for low VCT acceptability [30], [31], and need careful consideration when VCT is offered. Noticeably, the present study observed that alternative HIV testing strategies that are currently widely employed in China including community-based confidential and free mass HIV screening as well as an “opt-out” approach for HIV testing in various clinical settings were acceptable by most rural migrants. Future efforts should be tailored to improve the availability and utilization of these alternative HIV testing approaches among rural migrants.

The study has several limitations. First, the data were mainly self–reported and subject to recall or reporting bias. To minimize such bias, local experienced public health providers were trained to be interviewers and all interviews was anonymous and took place in a private space. Second, the effectiveness of the intervention program was assessed by repeated cross–sectional surveys among two independently selected samples but not a longitudinal cohort. However, a longitudinal cohort analysis might be least feasible in such big communities and particularly difficult to be stably maintained for rural migrants who were under frequent movement. Some researchers even argue that repeated cross sectional analyses are more appropriate than cohort analyses for measuring the effectiveness of community-based interventions [32]. Third, there were significant differences between the baseline and the follow-up surveys in terms of sociodemographic characteristics of study participants. These differences might be of influence on the effect evaluation. To address this problem, a multiple logistic regression analysis with adjustment of potential confounding effects of sociodemographic characteristics was performed to examine determinants of VCT acceptance after the intervention among participants in the follow-up survey. It indicated that out of the sociodemographic characteristics, working venue was independently associated with VCT acceptance among rural migrants.

In conclusion, community mobilization is critical to promotion and acceptance of HIV testing and counseling as well as promotion of risk awareness of HIV and safer sex among rural migrants in metropolitan areas in China. A comprehensive strategy with integration of VCT service and other HIV testing approaches is strongly necessitated to promote HIV testing and identification for the as large as 200 millions rural migrant population in China.
